# EARLY ORAL FEEDING POST-UPPER GASTROINTESTINAL TRACT RESECTION AND PRIMARY ANASTOMOSIS IN ONCOLOGY

**DOI:** 10.1590/0102-672020180001e1359

**Published:** 2018-06-21

**Authors:** Lilian Pinheiro LOPES, Taysa Machado MENEZES, Diogo Oliveira TOLEDO, Antônio Talvane Torres DE-OLIVEIRA, Adhemar LONGATTO-FILHO, José Eduardo de Aguilar NASCIMENTO

**Affiliations:** 1 Nutrition Department; 2 Upper Digestive Department at Barretos Cancer Hospital, Barretos, SP;; 3 Intensive Care Department at Albert Einstein Israelite Hospital, São Paulo, SP;; 4 Medical School, Univag, Varzea Grande, MT;; 5 Laboratory of Medical Investigation (LIM) 14, Faculty of Medicine, University of São Paulo, São Paulo, SP, Brazil.

**Keywords:** Esophagectomy, Gastrectomy, Surgical oncology, Feeding, Alimentação, Esofagectomia, Gastrectomia, Neoplasias gástricas, Neoplasias esofágicas

## Abstract

**Background:**

The practice of starving patients in the immediate period after upper gastrointestinal surgery is widespread. Early oral intake has been shown to be feasible and may result in faster recovery and decrease length of hospital.

**Aim:**

To evaluate the feasibility and safety of oral nutrition on postoperative early feeding after upper gastrointestinal surgeries.

**Methods:**

Observational cohort design study with convenience retrospective data in both genders, over 18 years, undergoing to total gastrectomy and/or elective esophagectomy. They have received oral or enteral nutrition in less than 48 h after surgery, and among those who started with enteral nutrition, the oral feeding up to seven days.

**Results:**

The study was performed in 161 patients, 24 (14.9%) submitted to esophagectomy, 132 (82%) to total gastrectomy and five (3.1%) to esophagogastrectomy. Was observed good dietary acceptance and low percentage (29%) of gastrointestinal intolerances, more pronounced among those with enteral diet. Most of the patients did not present postoperative complications, 11 (6.8%) were reopened, five (3.1%) had fistulas, three (1.9%) wound dehiscence, three (1.9%) fistula more wound dehiscence and six (3.7%) other non-infectious complications.

**Conclusion:**

Early oral diet is safe and viable for patients undergoing upper gastrointestinal surgery.

## RESUMO

**Racional:**

Os níveis do antígeno carcinoembriônico no lavado peritoneal têm sido demonstrados como possível fator prognóstico de recidiva e mortalidade em pacientes com câncer gástrico.

**Objetivos:**

Medir seus níveis em lavado peritoneal durante operação de ressecção de câncer gástrico e ver se eles aumentados estão relacionados com mortalidade, recorrência, recidiva e outros fatores prognósticos.

**Métodos:**

Foi realizado lavado peritoneal durante ressecções de câncer gástrico; os níveis do antígeno carcinoembriônico nesse lavado maiores ou iguais a 210 ng/g foram considerados aumentados ou positivos.

**Resultados:**

Foram estudados 30 pacientes, destes, 33,33 % apresentaram níveis aumentados, os quais foram fator de risco para mortalidade em seis meses OR: 8,5 (1,458-49,539) IC 95%, mortalidade geral RR: 2,111 (1,314-3,391) IC 95%, mortalidade devido à doença OR: 12 (1,885-76,376) IC 95%, recorrência peritoneal OR: 9 (1,325-61,138) IC 95%, e recidiva ou progressão da doença OR: 27 (2,705-269,460) IC 95%.

**Conclusões:**

Os níveis aumentados do antígeno carcinoembriônico no lavado peritoneal foram fatores de risco para mortalidade, recorrência peritoneal, recidiva e progressão da doença em pacientes com câncer gástrico.

## INTRODUCTION

Cancers of the upper gastrointestinal tract are among those with the highest prevalence and mortality in the world. In 2012, a global incidence of 455,000 esophageal and 951,000 stomach cancer cases were estimated, with mortality rates of 400,000 and 723,000 cases respectively^16^. According to data from INCA (José Alencar Gomes da Silva National Cancer Institute), in 2016, 12,920 new cases of stomach cancer in men and 7,600 in women were expected in Brazil and for esophageal cancer 7.950 in men and 2,860 in women^4^. 

Malnutrition in cancer patients is of serious concern; evidences show that 8-84% of patients would suffer from malnutrition in the course of the disease^2^. At the time of diagnosis, these patients were already considerably malnourished. Malnutrition actually worsens upon the inclusion of the different treatments (chemotherapy, radiotherapy and operation), further worsening the patient’s general condition. It is paramount that adequate nutritional needs be met before, during and after treatment^13^.

Nutritional therapy for malnourished patients has shown benefits, which have been translated into better tolerance to therapeutic interventions, reduction in hospital stay, better quality of life, and reduction of postoperative morbidity and mortality^7^.

Before such surgeries are carried out, oral feeding restriction over several days is widely practiced. The reluctance to allow early oral feeding is based on the concern over gastric distention and integrity of the anastomosis. On the other hand, over the last decades, studies have reported benefits in early feeding within a maximum of 48 h interval in the postoperative period for patients submitted to esophageal^10^ and gastric resections^5^^,^ 7 ^,^ 12. Benefits of this procedure are: scar and splanchnic flow improvement; stimulation to intestinal motility, reducing stasis; reduction on the incidence of infectious complications; decreased length of hospital stay; reduction of morbidity and mortality, culminating in cost reduction^7^.

Early oral feeding stimulation has been shown to be feasible and may result in a more rapid recovery of bowel function and a decrease in hospital stay, as well as not increase in morbidity when compared to oral feeding retention with jejunostomy during the first five postoperative days, when submitted to surgeries of the upper gastrointestinal tract ^8)^.

The goal of this study is to analyze the impact and safety of early oral feeding after esophagectomy and total gastrectomy.

## METHODS

Observational study of a cohort design with retrospective collection of patients submitted to total gastrectomy and/or esophagectomy at Barretos Cancer Hospital, Barretos, SP, Brazil, from January 2011 to December 2014. Sampling was done by convenience, and the information analyzed was obtained from routine data from the nursing ward and the outpatient unit, collected by the hospital nutrition department and complementary data gathered from medical records.

Data assessed were: information comprising identification, diagnosis, TNM classification of malignant tumors (TNM), physical status classification according to the American Society of Anesthesiology (ASA), chemotherapy and/or neoadjuvant radiotherapy, postoperative complications (fistula, surgical reoperation and dehiscence) and diet (gastrointestinal intolerance), length of hospital stay, hospital readmission, anthropometric assessment (weight, height, usual weight) and nutritional therapy initiated up to the 7^th^ postoperative day (enteral and oral routes), as well as its acceptance.

Patients included in the study were aged 18 years and older, of both genders, which underwent elective total gastrectomy and/or esophagectomy, received oral or enteral diet in less than 48 h postoperatively. Among patients who started the postoperative oral feeding by enteral catheter, those who started oral feeding within seven postoperative days were included in the study. Patients who did not receive an enteral diet within 48 h or those who received oral feeding after seven days were excluded.

Diets were followed for the first seven days of hospitalization, even if the patient had an oral feeding supplemented with enteral. Only the oral route was considered as the main route or if the patient had an enteral associated with parenteral nutrition, only the enteral was considered as the main route.

### Statistical analysis

Data were tabulated and analyzed using SPSS Statistics 21.0^®)^ software. Descriptive analyzes such as frequencies, dispersion and central trend measures were performed according to the characteristic of the variable (qualitative or quantitative) and graphs and tables were generated to better interpret the descriptive results. Univariate analyzes were performed, in a first moment, to compare the variable responses of interest in the study, with the other variables. Using a significance level of 5%, initially, the chi-square or Fisher’s test was performed. A logistic regression model was used to verify if there was a relationship between the variable of interest and the rest of the study. Variables with a level of significance of up to 20%, in the univariate analyzes, entered into the selection of logistic regression variables.

## RESULTS

The study was carried out with 161 patients, of which 24 (14.9%) were submitted to esophagectomy, 132 (82%) underwent total gastrectomy and five (3.1%) to esophagectomy associated with total gastrectomy; most of participants were men (n=111, 68.9%) with a mean age of 58±10.7 years. In relation to staging, those classified as T4 (n=54, 33.5%) and T3 (n=52, 32.3%) were predominant; sixty participants (37.3%) were N0 and 149 (92.5%) M0. One hundred cases (62.1%) were in ASA II surgical risk classification, and 107 (66.5%) were referred to the intensive care unit in the immediate postoperative period. One hundred and forty-eight patients (91.9%) had not undergone chemotherapy and 152 (94.4%) also did not undergo neoadjuvant radiotherapy.

Among all, 125 (77.6%) underwent preoperative nutritional assessment. The majority of them (n=82, 50.9%) was classified as eutrophic according to the classification of the body mass index according to age. Although considered to be eutrophic by BMI, 89 (55.3%) had weight loss, where 34 (38.2%) with the percentage of weight loss considered serious according to classification by Blackburn, Harvey^1^. The usual average weight was 72.7±16.85 kg, and the mean current weight (the last preoperative weighing obtained from the medical record) was 67.3±16.44 kg. The mean postoperative fasting time was 30.34±11.53 h.

**FIGURE 1:**
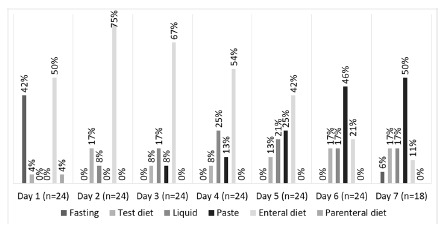
Evolution of post-esophagectomy nutritional therapy

 Patients who underwent esophagectomy (Figure 1), in the 1^st^ PO (n=24), 50% started enteral nutrition, 4% the test diet (water, tea and gelatin) oral intake and 42% were fasted. On the 2^nd^ PO, 25% were provided with a test diet or oral liquid diet and 75% enteral nutrition. On the 3^rd^ PO, the majority (67%) still received enteral nutrition as main route. This rate was reduced to only 11% in the 7^th^ PO, the oral feeding prevailing to 84% on that date.

**FIGURE 2:**
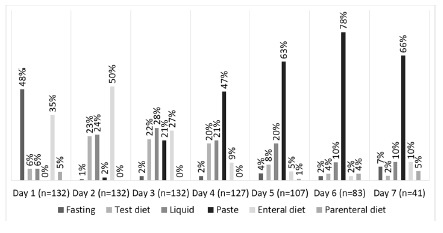
Evolution of nutritional therapy after total gastrectomy

Of the 132 (82%) patients submitted to total gastrectomy, 16 (12%) started the test diet or oral liquid diet on the 1^st^ PO, 46 (35%) started enteral nutrition and 64 were fasted (48%). On the 2^nd^ PO, 65 (49%) were receiving oral feeding and 66 (50%) via enteral. On the 3^rd^ PO, 94 (71%) received an oral feeding and only 35 (27%) enteral nutrition. On the 7^th^ PO, 32 (78%) were already receiving oral feeding and only four (10%) maintained enteral nutrition (Figure 2).

**FIGURE 3:**
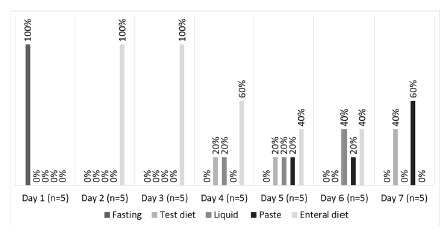
Evolution of nutritional therapy after esophagogastrectomy

The five patients who underwent esophagectomy associated with total gastrectomy (Figure 3) on the 1^st^ postoperative day were fasted. However, on the 2^nd^ day, an enteral diet was exclusively started for all patients and maintained on the 3^rd^ day, having the oral feeding started as of the 4^th^ day, and reaching the 7^th^ day with 100% of patients on an oral feeding as the main route.

As for the acceptance of the oral or enteral diet by the patients in relation to gastrointestinal intolerances (Table 1), it was possible to observe that they had good acceptance and there was a low percentage (29%) of gastrointestinal intolerance, with abdominal distension and vomiting prevailing over the seven days; most symptomatic patients were on enteral nutrition.

**TABLE 1:** Acceptance of postoperative (PO) oral and enteral diets

		1^st^ PO (n=78)	2^nd^ PO (n=160)	3^rd^ PO (n=158)	4^th^ PO (n=154)	5^th^ PO (n=132)	6^th^ PO (n=108)	7^th^ PO (n=58)
Diet acceptance	Yes	98.7%	96.8%	98.7%	96.1%	92.4%	94.4%	91.4%
	No	1.3%	3.1%	1.3%	3.9%	7.6%	5.6%	8.6%
Reason for non acceptance	Vomit	0%	0%	0%	1.3%	5.3%	2.8%	3.5%
	Diarrhea	0%	0%	0.6%	0%	0%	0%	1.7%
	Abdominal distension	1.3 %	3.1%	0.6%	1.9%	2.3%	2.8%	1.7%

In spite of the gastrointestinal intolerance, the majority of patients did not have their oral feeding suspended for a period over 24 h. Only 15 (9.3%) were suspended at some time during the 7-day stay.

According to Table 2, most patients did not present postoperative complications. Only 11 (6.8%) were reoperated in the current hospitalization; five (3.1%) had fistulas, three (1.9%) wound dehiscence, three (1.9%) fistula together with wound dehiscence and six (3.7%) other non-infectious complications. In a period of 30 days after hospital release, 11.8% were hospitalized again, with five (3.1%) due to surgical complications and 14 (8.7%) because of clinical problems. There were 9.9% of surgical reoperations after 30 days, infection in 15.7% with wound infection in 18 (11%). As a final outcome, 96.3% (n=155) were released from hospital and 3.7% (n=6) died.

**TABLE 2:** Postoperative complications and hospital outcome

Postoperative complications			
		n	%
Readmission to ICU during current hospitalization	No	153	95.0
	Yes	8	5.0
Reoperation during current hospitalization	No	150	93.2
	Yes	11	6.8
Non-infectious complication during hospitalization	No	144	89.4
	Yes	17	10.6
Non-infectious complication presented during hospitalization	Fistula	5	3.1
	Wound dehiscence	3	1.9
	Fistula + wound dehiscence	3	1.9
	Other	6	3.7
Rehospitalization in 30 days	No	142	88.2
	Yes	19	11.8
Reason for rehospitalization	Surgical intercurrence	5	3.1
	Clinical intercurrence	14	8.7
Reoperation within 30 days after first hospitalization	No	145	90.1
	Yes	16	9.9
Infection	No	134	84.3
	YEs	25	15.7
Type of infection	Wound	18	11.3
	Other	7	4.4
Outcome	Hospital release	155	96.3
	Death at ICU	6	3.7

ICU=intensive care unit

Simple analyzes were performed by way of the chi-square, relating noninfectious complications and the length of hospitalization with variables of interest. It was possible to realize that for the non-infectious complications the ASA classification was significant (p<0.05), since those with ASA III were the ones with a higher level of complications. The other variables were tested and no significant statistical difference was noticed (Table 3).

**TABLE 3:** Odds Ratio estimates of non-infectious complications, length of hospitalization and oral feeding

Variable	Category	Odds Ratio (OR)	95% C.I.		p-value
			Low	High	
ASA	I	1			.005
	II	5.656	.699	45.754	.104
	III	31.919	3.163	322.091	.003
Pre surgery nutritional assessment	No	1			
	Yes	.287	.092	.897	.032
Constant		.050			.005
Starting day for oral feeding postoperative (PO)		Odds Ratio (OR)	95% C.I.		p
			Low	High	
1st PO		1			.034
2nd PO		2.476	.799	7.676	.116
3rd PO		12.889	2.307	72.016	.004
4th PO		3.407	.920	12.620	.066
5th, 6th and 7th PO		1.511	.441	5.179	.511
Constant		1.125			.808

OI=oral intake; CI=confidence interval

As for the type of diet started on the first three postoperative days (oral or enteral), those who started with oral feeding 93 (64.6%) did not present complications, while 10 (58.8%) had non-Infectious complications. Those who started with an enteral diet, 51 (35.4%) presented no complications, while seven (41.2%) had non-infectious complications.

The variable on what postoperative day the oral feeding started did not present statistical difference. However, there was a trend towards a largest non-infectious complication in patients who started oral feeding after the 5^th^ PO (n=5, 29.4%), while only 22 (15.3%) did not have complications.

By way of the logistic regression test applied for non-infectious complications, it was verified (Table 3) that patients with ASA III surgical classification were at high risk (OR 32 CI 95% 3.1-322) compared to ASA I. It was also possible to observe that patients who have been through preoperative nutritional assessment had a 28.7% higher risk of having complications compared to those who did not, exhibiting the nutritional evaluation as a protective factor for non-infectious complications.

Another logistic regression analysis was performed relating the length of hospital stay and days of onset of oral feeding in the postoperative period (Table 3). Patients who started the diet on the 3^rd^ PO presented a 13-fold greater risk (OR: 12.8) for longer hospitalization than those who started the oral feeding on the 1^st^ PO (p<0.05). The mean overall hospital stay was 8.7 days.

## DISCUSSION

The current practice related to the early onset of oral nutrition in the postoperative period was well established for several abdominal surgical procedures. Some randomized controlled trials and meta-analyzes have shown that the early initiation of oral feeding is feasible and safe after upper gastrointestinal surgeries, and suggest that this practice may reduce infection related to potential complications and length of hospital stay compared with the traditional approach “nothing by the mouth”^3^^,^ 6 ^-^ 9 ^,^ 15.

In the present study, all patients started some kind of oral feeding (test, liquid or paste) within seven days, and up to 48 h after surgeries, some started oral or enteral diet with good acceptance; even setting the operations apart, it can be verified that in both gastrectomy and esophagectomy, oral feeding started 48 h after surgery in some patients, and only in the esophagogastrectomy there was a bigger concern before starting it. Although oral and enteral diet values ​​were very close and there was no statistically significant difference, there was a trend showing that enteral diet patients had a greater chance of noninfectious complication.

A study by Jo et al^6^ with 132 patients submitted to gastrectomy for gastric adenocarcinoma, 89% started oral water intake on the 1^st^ PO and a light diet with good acceptance after 3^rd^ PO. In the meta-analysis by Liu et al^9^, six studies were compared in which patients underwent some type of gastrectomy. They were divided into two groups, where the majority started on an early oral feeding (water or other liquids) on the same of the surgery or on the 1^st^ day after the surgery, and about 90% of the patients responded well. In this study we evaluated the acceptance of diets and gastrointestinal symptoms both for patients who were receiving oral and early enteral diets. In general, there was good acceptance during the first seven days of hospitalization, when the main gastrointestinal symptoms displayed were abdominal distension and vomiting, which were observed more frequently in patients receiving an enteral diet.

Furthermore, the present study showed low values ​​of postoperative complications. When analyzing non-infectious complications in general, no significant statistical association was found with the early initiation of oral or enteral diet, or directly related to the day the oral feeding was started. Statistical analysis was associated with ASA classification as a risk factor, and pre-surgical nutritional assessment was shown to be a protective factor against noninfectious complications.

Sierzega et al^14^ also found low rates of surgical complications in their study; in the group of patients who received an early oral feeding there was a 15% rate compared to 24% of general surgical complications with a significant statistical difference; among these complications wound infections (12%) predominated in both groups; there were 11 reoperations in each group, and mortality of five patients in the early oral feeding group and six in the late feeding group.

According to the study by Lassen et al^8^, which separated patients submitted to several different types of upper gastrointestinal operations in one enteral diet group and one oral feeding group - where both groups started dieting on the 1^st^ postoperative day - those who received the enteral diet had greater surgical complications than the oral feeding group with a statistically significant difference. Reoperation cases were 15.9% for the enteral diet group and 13.2% for the oral group; wound infection was 8.8% vs. 5%; mortality after 8 weeks of 8.4% vs. 5.9%.

Pan et al^11^ showed that the incidence of complications was 29% in patients of the fast-track group and 40% in the conventional group. There were no significant differences in the incidence of complications, readmission rate at 30 days or reoperation between the two groups within 30 days after esophagectomy. No patient died in either group.

Thus, it was possible and safe to feed patients who underwent total gastrectomy or early oral or enteral esophagectomy postoperatively. Furthermore, mortality was low and there was no higher incidence of fistula in the upper tract. However, the limitation of the present study is that it is observational, retrospective and unicentric. 

## CONCLUSION

Early oral feeding is safe and feasible for patients undergoing total gastrectomy and/or esophagectomy. In addition, there is a positive impact in reducing hospital stay in those who started an early oral feeding, that is, on the 1^st^ PO.

## References

[1] Blackburn GL, Harvey KB (1982). Nutritional assessment as a routine in clinical medicine. Postgrad Med.

[2] Chaves MR, Boléo-tomé C, Monteiro-grillo I, Camilo M, Ravasco P (2010). The Diversity of Nutritional Status in Cancer New Insights. Oncologist.

[3] Hosseini SN, Mousavinasab SN, Rahmanpour H, Sotodeh S (2010). Comparing early oral feeding with traditional oral. Turkish J Gastroenterol.

[4] Instituto Nacional de Cancer José Alencar Gomes da Silva (2015). INCA - Instituto Nacional de Câncer - Estimativa 2016 [Internet].

[5] Jiang W, Zhang J, Geng Q, Xu X, Lv X, Chen Y (2016). Early enteral nutrition in neonates with partial gastrectomy A multi-center study. Asia Pac J Clin Nutr.

[6] Jo DH, Jeong O, Sun JW, Jeong MR, Ryu SY, Park YK (2011). Feasibility Study of Early Oral Intake after Gastrectomy for Gastric. Carcinoma.

[7] Laffitte AM, Polakowski CB, Kato M (2015). Realimentação precoce via oral em pacientes oncológicos submetidos à gastrectomia por câncer gástrico. ABCD Arq Bras Cir Dig.

[8] Lassen K, Kjæve J, Fetveit T, Tranø G, Sigurdsson HK, Horn A (2008). Allowing Normal Food at Will After Major Upper Gastrointestinal Surgery Does Not Increase Morbidity. Ann Surg.

[9] Liu X, Wang D, Zheng L, Mou T, Liu H, Li G (2014). Is Early Oral Feeding after Gastric Cancer Surgery Feasible?. A Systematic Review and Meta-Analysis of Randomized Controlled Trials.

[10] Mahmoodzadeh H, Shoar S, Sirati F, Khorgami Z (2015). Early initiation of oral feeding following upper gastrointestinal tumor surgery a randomized controlled trial. Surg Today.

[11] Pan H, Hu X, Yu Z, Zhang R, Zhang W, Ge J (2014). Use of a fast-track surgery protocol on patients undergoing minimally invasive oesophagectomy?: preliminary results.

[12] Selby L V., Rifkin MB, Yoon SS, Ariyan CE, Strong VE (2016). Decreased length of stay and earlier oral feeding associated with standardized postoperative clinical care for total gastrectomies at a cancer center. Surgery (United States).

[13] Shoar S, Naderan M, Mahmoodzadeh H, Hosseini- N, Mahboobi N, Sirati F (2016). Early Oral Feeding After Surgery for Upper Gastrointestinal Malignancies A Prospective Cohort Study. Oman Med J.

[14] Sierzega M, Choruz R, Pietruszka S, Kulig P, Kolodziejczyk P, Kulig J (2015). Feasibility and Outcomes of Early Oral Feeding After Total Gastrectomy for Cancer.

[15] Weijs TJ, Nieuwenhuijzen GAP, Ruurda JP, Kouwenhoven EA, Rosman C, Sosef M (2014). Study protocol for the nutritional route in oesophageal resection trial: a single-arm feasibility trial (NUTRIENT trial). BMJ Open.

[16] WHO (2012). Globocan [Internet].

